# A protocol for the *HeadCoach* trial: the development and evaluation of an online mental health training program for workplace managers

**DOI:** 10.1186/s12888-018-1603-4

**Published:** 2018-01-29

**Authors:** Aimée Gayed, Bridget T. Bryan, Katherine Petrie, Mark Deady, Allison Milner, Anthony D. LaMontagne, Rafael A. Calvo, Andrew Mackinnon, Helen Christensen, Arnstein Mykletun, Nicholas Glozier, Samuel B. Harvey

**Affiliations:** 1 School of Psychiatry, University of New South Wales, Randwick, Australia; 2 Black Dog Institute, Randwick, NSW Australia; 3 School of Population and Global Health, The University of Melbourne, Melbourne, Australia; 4 Centre for Population Health Research, Deakin University, Geelong, VIC Australia; 5 School of Electrical and Information Engineering, University of Sydney, Sydney, Australia; 6 Centre for Mental Health, University of Melbourne, Melbourne, Australia; 7 Department of Mental Health and Suicide, Norwegian Institute of Public Health, Oslo, Norway; 8 Department of Community Medicine, University of Tromsø, Tromsø, Norway; 9 Centre for Work and Mental Health, Nordland Hospital Trust, Bodø, Norway; 10 Centre for Research and Education in Forensic Psychiatry and Psychology, Haukeland University Hospital, Bergen, Norway; 11 Brain and Mind Centre, Sydney Medical School , University of Sydney, Australia

**Keywords:** Manager, Supervisor training, Workplace mental health, Mental health education, Online intervention, Randomised control trial, Knowledge, Attitudes, Behaviour, eHealth

## Abstract

**Background:**

Within high income countries, mental health is now the leading cause of long term sickness absence in the workplace. Managers are in a position to make changes and decisions that have a positive effect on the wellbeing of staff, the recovery of employees with mental ill health, and potentially prevent future mental health problems. However, managers report addressing workplace mental health issues as challenging. The aim of the *HeadCoach* trial is to evaluate the effectiveness of a newly developed online training intervention to determine whether it is able to build managers’ confidence to better support individuals within their teams who are experiencing mental ill health, and the confidence to promote manager behaviour likely to result in a more mentally healthy workplace.

**Methods/Design:**

We will conduct a cluster randomised control trial (RCT) to evaluate the effect of *HeadCoach*, an online training intervention for managers with a focus on the mental health of their employees, compared to a waitlist control. The target sample is 168 managers, and their direct employees. Managers and employees will be assessed at baseline and at 4-month follow up. Managers will have an additional, intermediate assessment 6-weeks post-baseline. The primary outcome is change from baseline in managers’ self-reported confidence when dealing with mental health issues within their team and promoting a mentally healthy workplace. The difference between the intervention and waitlist control groups will be assessed using linear mixed effects repeated measures (MMRM) analysis of variance (ANOVA). Secondary managerial outcomes include mental health literacy, attitudes towards mental health issues in the workplace and managerial behaviour in dealing with mental health matters with their staff. Employee outcomes will be perceived level of manager support, engagement, psychological distress, and rates of sickness absence and presenteeism.

**Discussion:**

To our knowledge this will be the first RCT of a purely online training intervention developed specifically for managers that promotes confidence to both support staff experiencing mental ill health and create a mentally healthy work environment. If successful, this intervention has the potential to provide an effective and efficient method of training managers in workplace mental health and to enhance employee wellbeing.

**Trial Registration:**

Australian and New Zealand Clinical Trials Registry ACTRN12617000279325

## Background

Mental ill health has become the leading cause of long-term sickness absence and work incapacity in many high income countries [1–3]. The most common mental conditions in the workplace are treatable and often preventable conditions including depression, anxiety, and stress-related disorders [4–6]. For some workers, the development or persistence of their mental health condition may, in part, be related to their workplace [7]. Conflicting and excessive work demands, a lack of job control, and poor collegial relationships and support have all been identified as primary sources of work-related stress that can impact upon employees’ well-being and productivity [8]. Many of these risk factors have the potential to be influenced by managers or other supervisors in the workplace. Positive leadership behaviour has also been identified as reducing the risk of sickness absence and as one of the determinants of employees returning to work following a period of sickness absence [1, 9].

Managers are in a unique position to manage work-based mental health risk factors and improve the mental health of their workers. Their knowledge of workplace issues, their ability to implement adjustments to working conditions, and their position to lead by example with regards to acceptance and understanding of mental health issues can influence changes and decisions to benefit their workers. As a result, managers may be able to implement reactive and preventative managerial strategies through primary, secondary and tertiary interventions as a means to enhance their employees’ mental health and to aid the recovery process of those workers who have become unwell [10, 11].

Best practice guidelines are available outlining the role managers should play in sickness absence, regardless of the underlying cause of the illness [12, 13]. These include, but are not limited to, behaviours that facilitate regular conversations with an employee, maintaining a focus on the employee’s well-being and being able to develop an appropriate return to work plan. However, managers often report feeling unsure what to do when a staff member is ill, particularly if they are suffering from a mental illness [5, 13]. Guidance is also available detailing how managers can modify their behaviour and the overall psychosocial work environment in order to reduce mental health risk factors for their staff [13, 14]. In line with this, agencies such as the Health and Safety Executive in the UK have provided management standards and descriptions of good practice across six areas of work that can impact negatively on employee health if not managed properly [14]. These areas of work design concern demands of the job, having a sense of control of duties, availability of workplace support, role clarity, communication within the organisation, and the promotion of positive working relationships. By effectively managing these primary sources of stress at work, managers can promote high level of health, well-being and organisational performance. However, once again, it is not clear how many managers utilise this advice and many report feeling unsure of how to best implement these ideas [5, 13].

A small body of research, comprising a number of pilot studies and a few small scale controlled trials, has evaluated specialised training programs for managers to promote understanding of mental health problems among workers, with evidence suggesting that managers value and benefit from such initiatives [2, 3, 15–17]. However, other controlled trials have not found any positive effects in terms of changing the attitudes of managers [11] and have failed to find any evidence that mental health training for managers has an impact on either their managerial behaviour of mental health matters or on objective measures of mental health amongst their direct reports [18, 19].

There is increasing recognition that best practice in workplace mental health requires an integrated approach that prevents harm, promotes positive mental health, and addresses mental health in the workplace regardless of the cause of the illness [8]. However, to date, the way in which these key components can be delivered together through manager training has not been well-articulated, resulting in either a lack of manager education or separate uncoordinated educational programs.

The current trial will test an integrated and comprehensive online training intervention for managers called *HeadCoach.* The use of online technology through a mobile and desktop compatible website will provide a number of benefits compared with traditional modes of workplace training [20]. Although there is limited personal interaction with educators and other participants, which can be a key component to effective learning, web-based training offers a greater deal of flexibility which is suitable for organisational based learning. It reduces the time commitment and impact on organisational resources by removing factors such as time taken to travel to and from training and the impact of having a large number of managers away from their jobs simultaneously. Online training allows participants to schedule training around the demands of their job, and course material can be revisited as required providing a greater opportunity for consolidation of content.

*HeadCoach* will be delivered to managers with a focus on how to best support the mental health needs of employees reporting directly to them. The primary aim of this trial is to evaluate the effectiveness of the *HeadCoach* intervention to improve managers’ confidence to effectively respond to the needs of staff experiencing mental health issues and to implement evidence-based managerial techniques that promote a more mentally healthy workplace.

## Methods/Design

### Study design

*HeadCoach* will be evaluated via a cluster randomised controlled trial (RCT) conducted within three industry partner organisations. Clusters will comprise of groups of managers based on organisational-specific pre-existing geographical work zones to reduce the risk of contamination between the intervention and control groups. Stratification will occur based on the number of managers within each zone for each industry. A researcher independent from the trial will use a computer-generated program to conduct the stratified randomisation of workplace zones prior to recruitment. Outcome data will be collected from the managers and from employees who report directly to them.

### Intervention

The *HeadCoach* online training intervention aims to build managers’ confidence in dealing with mental health issues in the workplace. This includes managers’ confidence when responding to mental illness and/or distress amongst their staff, and the implementation of behaviours supporting more mentally healthy workplaces to help prevent new mental health problems. Building confidence across these reactive and preventative managerial strategies to better manage mental health issues in the workplace is a key step in altering managers’ behaviours across these domains [21]. Improving confidence is also one of the central concepts of Self-Efficacy Theory, which suggests that people are more likely to engage in particular behaviours across a range of settings when they feel more capable of successfully attaining the intended outcome [22].

The intervention is self-guided and divided into three broad topics to be completed sequentially. The first topic, *Common mental illnesses*, provides an introduction to mental health issues commonly found in the workplace. The next topic, *How to help an employee you are concerned about*, discusses ways managers can identify people within their team who may be at risk of mental health issues, how managers can support their workers, and direction around how to best have a conversation with employees who may be experiencing mental ill health. It also discusses how to help employees stay at work or return to work faster following a period of sickness absence due to mental illness. The third and final topic, *Minimising mental health risks in the workplace,* examines managerial skills that are useful in reducing mental health risk factors in the workplace in order to create a mentally healthy workplace for all employees.

Each topic comprises between three and seven 10-min modules featuring text, activities, short videos, and practical exercises for individuals to complete. The course outline listing the twelve modules, three topic consolidation exercises and three topic toolboxes providing quick access to useful resources presented in that topic, is outlined in Table 1. Depending on individual learning styles, the entire program is expected to take approximately 2.5 hours to complete. It has been designed so managers can work through the content at their own pace across a 6-week period. The intervention will be delivered online through a mobile responsive website and can be accessed using a desktop, laptop, tablet, or smart phone.

**Table 1:** Course outline for *HeadCoach*

*HeadCoach* Manager Training	
Topic 1: Common Mental Illnesses	
Module 1: Recognising Mental Health Issues	
Module 2: The Workplace and its People	
Module 3: Topic Summary Exercises	
Module 4: Topic Toolbox	
Topic 2: How to Help an Employee you are Concerned About	
Module 1: Identifying People at Risk	
Module 2: Providing Support	
Module 3: Having the Talk	
Module 4: Facilitating Help Seeking	
Module 5: Modifying Work to Help Recovery	
Module 6: Returning to Work	
Module 7: Topic Summary Exercises	
Module 8: Topic Toolbox	
Topic 3: Minimising Mental Health Risks in the Workplace	
Module 1: How to be a Respectful and Responsible Manager	
Module 2: Managing and Communicating Existing and Future Work	
Module 3: Managing Individuals within your Team	
Module 4: Managing Difficult Emotions	
Module 5: Topic Summary Exercises	
Module 6: Topic Toolbox	

### Participants

We aim to recruit both managers and their direct reports from three large industry partner organisations collaborating with this trial. These industry partners come from the emergency services and construction sectors. Together they have 690 managers and 9100 employees reporting directly to these managers who could potentially participate in the trial.

### Inclusion criteria

To be included in the study, participants must be 18 years or older; currently residing in Australia; have good English comprehension; and be a current employee of one of the three collaborating industry partners. In addition, participants in the manager group must supervise a team of three or more employees.

### Recruitment process

The process of recruitment will commence with each industry partner distributing an introductory email about the trial to managers at a certain level within each organisation and their direct employees describing the purpose of the research, outlining what is involved in the trial participation, and emphasising the voluntary nature of participation. It will discuss the proposed benefits of the trial to the individual and the industry, outline the independence of the research team from their employer, and advise that all components of the trial may be completed during work hours. Confidentiality of participation and data from their employer is assured.

Within the following week, all direct report employees of the managers within each cluster will receive an email from the research team inviting them to participate in the research study. This email will contain a link to the online research platform which employees can click to opt into the study and commence participation. At the completion of the recruitment period for direct report employees, participants at the managerial level will receive a manager-specific email invitation. This staggering of recruitment is designed to ensure that baseline employee data is obtained prior to the managers receiving any intervention. This email will invite managers to take part in the evaluation of an online educational training program about mental health in the workplace. As with the employee recruitment email, this email will include a link for managers to click if they choose to opt into the study.

### Trial procedure and assessment

**Fig. 1:**
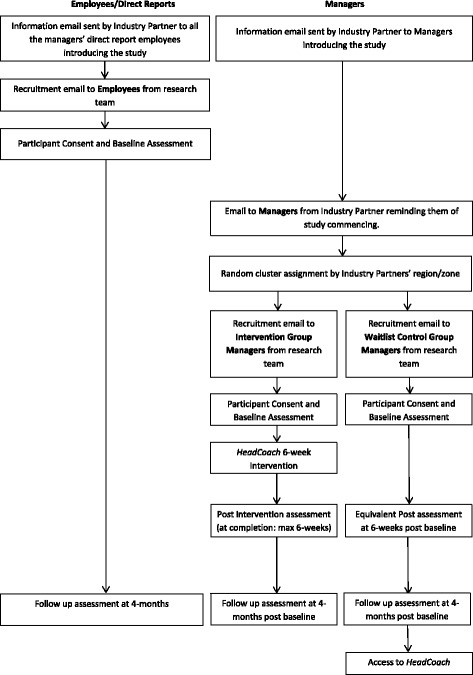
Participant flow-chart for the *HeadCoach* trial

Figure 1 outlines the trial procedure and stages of assessment for participants at the manager and employee levels.

#### Employees

Direct report employees who opt into the trial and provide consent via an online consent form will complete a 10-min baseline questionnaire. Demographic details, as well as information relating to own mental health, perceived level of manager support, self-reported absenteeism and presenteeism, psychosocial safety climate and work engagement will be collected. The completion of this baseline questionnaire will be the only requirement for employee level participants until the 4-month follow-up questionnaire. This follow-up questionnaire will be distributed by email and will contain the same assessment items as the baseline questionnaire. A unique code assigned to employee email addresses will be used to maintain participants’ privacy while allowing pre-post data to be linked. Employees who complete the 4-month employee questionnaire will indicate at the completion of the questionnaire whether they wish to be entered into a prize draw for one of three $250 vouchers by providing their name and contact details, with reassurances that this information will be kept separately from their responses and not disclosed to their employer.

#### Managers

At the completion of the baseline data collection period for employees, managers will be invited by email to participate in the study irrespective of whether their employees responded to the baseline questionnaire. Managers who agree to participate and provide online consent will create their individual *HeadCoach* registration account and complete a baseline assessment questionnaire. Demographic details and self-reported information on mental health literacy, attitudes towards mental health issues, confidence in dealing with mental health issues within the workplace, managerial practices used to support the mental health needs the team, and demographic details will be collected. Managers in the intervention group will then be directed into the online *HeadCoach* manager training program while those managers in the control group will be advised they will receive access to the training after a waiting period.

Post intervention questionnaires will be disseminated by email to the intervention and control groups at 6-weeks after completion of the baseline questionnaire. This 6-week period comprises the training period allocated to managers to complete *HeadCoach*. If a manager finishes all components of the online program earlier in the 6-week training period, they will be invited to complete the questionnaire at that earlier point of completion. This approach is expected to prevent non-response and ensure an accurate measure of the post intervention effect.

At the 4-month post-baseline assessment, a final data collection for the primary outcomes and other measures will be conducted for all managers and employees participating in the trial. An email will be sent to all manager and employee level participants inviting them to complete the final questionnaire, and to advise of a prize draw offered for its completion. Managers who complete the 4-month post questionnaire will be entered into a separate prize draw from the employees, for one of three $250 vouchers. Following the completion of all data collection, managers in the waitlist control group will be provided with online access to the *HeadCoach* program.

### Outcome measures

Managers and employees will complete separate questionnaires at each time point. Data will be collected via a secure, encrypted online research platform and de-identified to maintain the privacy of participants.

#### Primary outcome

The primary outcome is managers’ self-reported confidence when responding to staff members experiencing mental health problems and when considering how to alter the workplace environment in order to enhance employee mental health and well-being. Managers’ level of confidence will be assessed at 6-week and 4-month follow-ups, with the primary outcome being considered at 4-months post baseline. Confidence in managing mental health issues and promoting a mentally healthy workplace will be measured using a modified version of a previously published supervisor scale [10]. This scale describes six workplace scenarios and asks managers to indicate their level of confidence in dealing with each of these, on a five-point Likert scale ranging from *not at all* to *extremely confident.* This results in an overall confidence score ranging from 6 to 30. The types of scenarios asked about include “Initiating contact with staff on sickness absence leave that you believe might be due to mental illness” and “Creating a work environment that prevents and reduces stress within my team”*.* The wording of these vignettes aims to focus on managers’ self-efficacy and perceived confidence. As noted above, self-efficacy and confidence are thought to be key determinants of behaviour modification [22], with evidence suggesting that building confidence in managing workplace mental health problems can increase managers’ promotion of positive mental health within their teams [21].

#### Secondary outcomes

At each of the data collection points, information on a range of secondary outcomes will be assessed at the manager level. Measures that will be used to help understand the reasons for any shift in confidence include: knowledge about common mental health problems (measured using the Mental Health Knowledge Schedule (MAKS)) [23]; stigmatising attitudes towards mental illness (using a modified version of previously published personal stigma scales) [24–26]; and understanding of their role as a manager in dealing with mental health in the workplace [12]. Additionally, a range of secondary outcomes will be used to measure any changes in managers’ behaviour. Our ability to reliably measure managers’ behaviour in the workplace is limited by a number of key factors. By definition, a managers’ responsiveness to and support for staff experiencing mental health issues can only be measured once an employee develops and discloses mental health symptoms. It is likely that within a four-month trial, this would only occur for a minority of managers. This is the main reason that confidence regarding future behaviour is the primary outcome in this study. However, when a manager has experienced a staff member with mental health problems they will be asked how they responded. In addition, all managers will complete a questionnaire that seeks to enquire about their responsiveness to mental health problems in the workplace (for example “I initiate a conversation with individuals I supervise about their mental health and well-being”*)* with options on a five-point Likert scale ranging from *strongly disagree* to *strongly agree*. Managers will also be asked how often they apply a range of managerial techniques that promote a mentally healthy workplace using an adapted version of the HSE Management Standards Indicator Tool [14]. Finally, managers’ own level of psychological distress will be assessed with the Kessler Psychological Distress Scale (K6) [27].

Secondary outcomes will also be assessed at the employee level, although we acknowledge the possibility that any beneficial impact on employees may not be seen within the timeline of this type of study. Employees’ self-reported levels of well-being and psychological distress will be assessed by the 7-item Short Warwick-Edinburgh Mental Well-Being Scale [28] and the 6-item Kessler Psychological Distress Scale (K6) [27] respectively. Rates of absenteeism will be measured using employees’ self-report of recent sickness absence, and items from the World Health Organisation HPQ (WHOHPQ) Presenteeism Questionnaire [29] will be used to measure employees’ work performance. Employees’ perceived level of support from managerial staff will be measured through the Psychosocial Safety Climate Scale [30] and the manager scales on a workplace social support scale [31, 32]. Level of employee work engagement will be measured through a variation of the Utrecht Work Engagement Scale used in previously conducted work engagement surveys [33].

Demographic information including age, gender, job role and length of service in this role will also be collected for managers and employees.

### Statistical analysis

Primary analyses will be undertaken within an intent-to-treat framework, retaining all participants as randomised, regardless of extent of engagement with training or withdrawal from the study. Likelihood based methods (mixed-model repeated measures (MMRM)) will be used to assess significance of change in the two primary manager outcome measures. Clustering will be accommodated by a random cluster membership (work site) factor. A priori planned comparisons of change from baseline to the 4-month endpoint will test the primary hypothesis. An unstructured variance-covariance matrix will be used to accommodate relationships between observations at different occasions of measurement. Stratification variables and any variables found to be substantially imbalanced between intervention arms post randomisation will be tentatively included in these models and retained if statistically significant and influential on outcomes. Length of time from baseline to complete training and associated post-intervention assessment will also be introduced tentatively into models to evaluate the effects of adjustment for possible effects associated with some managers undertaking these assessments earlier than others and earlier than control group participants.

Similar analyses of scaled secondary manager outcomes will assess differential change due to intervention arm. Mathematical transformation or categorisation of raw scores may be undertaken to meet distributional assumptions and address any violation of assumptions attributable to outliers. Comparable analyses of employee measures will additionally accommodate the nesting of employees within managers by including a random effect for managers where this is possible.

Additional per-protocol analyses to assess effectiveness of the program will focus on managers who completed at least one of the three topics. This subgroup will be compared to all responders in the waitlist control group and, secondarily, with managers who completed less of the training material. The latter comparison will inform the dose-response profile of the intervention and, in particular, whether completing only a subset of the earlier components of *HeadCoach* has a benefit comparable to the complete program.

All tests of treatment effects will be conducted using a two-sided alpha level of 0.05 and 95% confidence intervals. A member of the research team who is blinded to the intervention and waitlist control allocations will perform this analysis. The nature of the intervention will make blinding managers to their group allocation impossible.

### Sample size and power calculations

Our industry partners comprise a total of 42 geographical clusters with an average of 20 managers in each cluster. Participation in this study will be voluntary. Based on pilot data, we assume a conservative participation rate of 20% of all managers, with a drop-out rate of 25%. Based on these assumptions, we anticipate being able to recruit a minimum of 168 managers and to successfully follow up 126. Assuming an intraclass correlation of 0.05 yields a design effect of 1.15, the forecast achievable sample size would have 80% power to detect a moderate effect size of 0.54 using an alpha value of 0.05.

## Discussion

This protocol describes a cluster RCT of an online workplace mental health training intervention developed specifically for managers. To the best of our knowledge, this will be the first evaluation of an online intervention that simultaneously addresses how managers can best support and respond to employees experiencing mental health issues, while also promoting primary prevention strategies that may be valuable in creating a mentally healthy workplace for the whole team. Workplace mental health interventions have been found to be effective for particular manager level outcomes such as mental health literacy and mental health promotion behaviours within the workplace [11], as well as improved recognition of and more positive attitudes towards mental health [34]. However, there is limited research with data obtained from both the manager and their direct reports examining the impact of building manager confidence. Data collected for this trial will enable evaluation of outcomes from both managers as well as from employees reporting to them over a four month follow up period.

The dissemination of workplace training via the internet can offer many potential benefits as it offers flexible, yet standardised delivery of educational material, the opportunity for revision of modules, reduced organisational expenses associated with staff attending face-to-face training, and the opportunity to monitor progress through the research platform [20]. However, reduced adherence and low program completion rates are potential problems associated with professional development delivered via eLearning tools. Managers may experience difficulties in prioritising online training sessions due to existing work demands and time pressures compared to a scheduled face-to-face learning session. These factors were taken into consideration when developing *HeadCoach* by designing the program to be easily integrated into a managers’ workload and own learning style. The twelve modules and three topic consolidation exercises are approximately 10 min each in duration. Managers’ may choose to complete one at a time, or to work through as many as they can depending on the time available to them, thus potentially increasing the opportunity for program adherence. Data on program adherence and engagement will be available through the research platform.

A cluster randomisation design has been selected for this RCT. Although this reduces the power of the sample, which has been estimated as capable of detecting a moderate effect size, the method of randomising clusters of managers based on their geographical location reduces the risk of intervention contamination within worksites. However, in the event of substantial movement of managers between work sites, such contamination may be unavoidable. Within the time frame of this study, such movement is unlikely, but demographic data obtained at baseline and follow up will allow the exploration of any organisational movement.

It is also recognised that assigning worksites to the waitlist control condition may also produce some problems. Level of interest and motivation to participate in the trial may wane over time for those in the waitlist control as a result of the delay in being accorded access to the program, and dropout rates have the potential to be higher in the waitlist control group, as incentive to respond to follow up questionnaires may be reduced.

A further potential limitation is the selection of a primary outcome based on self-report, particularly as confidence levels may be prone to a social desirability bias. Although confidentiality of data from participants’ employer is assured, some participants may still hold concerns that could influence their choice to respond, or bias their responses towards more favourable reporting of their level of confidence in the performance of certain managerial behaviours.

Given managers can play a key role in promoting the mental health and well-being of their staff, but that they often report uncertainty about how to best address these matters, it is critical that managers are upskilled and educated, through the latest evidence-based information, in how they can best identify and support the mental health needs of their staff. If effective, the *HeadCoach* online program may provide a practical and efficient method of training a large number of managers in best workplace mental health practices.

### Trial status

Recruitment commenced in 2017 from all three industry partners. Data collection is ongoing. It is anticipated that data collection will be finalised in early 2018, with data analysis due to be completed by mid-2018.
